# Case report of a cystic parathyroidal adenoma with rapid growth induced by cinacalcet

**DOI:** 10.1186/s12902-020-0532-7

**Published:** 2020-04-20

**Authors:** Christoph Werner, Amelie Lupp, Gabriele Mtuka-Pardon, Christof Kloos, Gunter Wolf, René Aschenbach, Anika Biermann, Martin Freesmeyer, Philipp Seifert

**Affiliations:** 1 Clinic of Internal Medicine III, Jena University Hospital, Am Klinikum 1, 07747 Jena, Thuringia Germany; 2 Institute of Pharmacology and Toxicology, Jena University Hospital, Jena, Germany; 3 Clinic of General, Visceral and Vascular Surgery, Jena University Hospital, Jena, Germany; 4 Department of Radiology, Jena University Hospital, Jena, Germany; 5 Institute of Pathology, Jena University Hospital, Jena, Germany; 6 Clinic of Nuclear Medicine, Jena University Hospital, Jena, Germany

**Keywords:** Head and neck, Parathyroid, Adenoma, Cyst, Hyperparathyroidism

## Abstract

**Background:**

Primary hyperparathyroidism is a rare condition of disease which can seldomly present as giant retrotrhyroideal cysts, complicating the localization of the adenoma to resect.

**Case presentation:**

A 56-year old female presented with hypercalcaemia of 3.38 mmol/L (2.2–2.65 mmol/L) and a history of breast cancer. A fast growing cystic parathyroidal adenoma was diagnosed by a multimodal approach including comprehensive diagnostic imaging (ultrasonography, scintigraphies, dynamic MRI) and cytopathological investigations after ultrasonography-guided puncture. The patient was cured by surgical extraction of the whole adenoma. In retrospect, the rapid growth was most likely induced by cinacalcet (initially 30 mg/d, later 60 mg/d) therapy which the patient received for few months only. Primary hyperparathyroidism was ascertained because surgical removal of the solitary adenoma cured the patient. Furthermore, there was no relevant renal insufficiency or history of prolonged calcium-level deregulation.

**Conclusions:**

This phenomenon of cystic degeneration of parathyroidal adenoma under therapy with cinacalcet has previously been described in secondary hyperparathyroidism, but not in primary hyperparathyroidism and should be considered in diagnostic approach.

## Background

Primary hyperparathyroidism is a rare condition of disease resulting from either hyperplastic or adenomatous parathyroid tissue. In our tertiary care center, we often see patients with parathyroid adenomas that are hard to localize, needing a comprehensive diagnostic approach. Cystic parathyroidal adenomas are a much rarer entity of this disease but have been described several times before. Also, giant volumes are reported and iPTH measurement in cystic fluid was found to be a useful method to confirm diagnosis [1–3].

Cinacalcet is a drug approved for the treatment of primary, secondary and malignant hyperparathyroidism. It decreases the release of parathormone through increasing sensitivity for calcium by allosteric modulation of the calcium-sensing receptor on the parathyroid cells.

## Case presentation

A 56-year-old female was referred to the endocrinological department of a university hospital with the diagnosis of hypercalcaemia. Due to mid-back pain osteodensitometry was done prior to referral revealing pronounced osteoporosis (T-Score − 4.2 BMD: 0.589 g/cm^3^). External laboratory diagnostics found hypercalcaemia, vitamin D deficiency and hyperparathyroidism. Total calcium was 3.38 mmol/L (2.2–2.65 mmol/L), ionized calcium 1.71 mmol/L (1.15–1.29 mmol/L), serum phosphate 0.72 mmol/L (0.76–1.37 mmol/L), vitamine D (25-OH) 66.1 nmol/L (75–375 nmol/L), vitamine D (1.25-OH) > 360 pmol/L (36.5–216.2), intact parathyroid hormone (iPTH) 190 ng/L (6.7–38.8 ng/L, assay listed in S1), thyroid stimulating hormone (TSH) 0.71 mU/L (0.25–4.04 mU/L), estimated glomerular filtration rate (eGFR) (CKD-EPI) 84 mL/min. There was no proteinuria.

Due to the history of breast-cancer diagnosed 4 years before and in complete remission since curative therapy (which was immediately initiated after diagnosis) metachronous bone metastases were excluded via MRI of the spine and breast as well as a scintigraphic bone-scan. Chronic and acute kidney-injuries, nephrolithiasis and nephrocalcinosis were excluded by kidney-ultrasonography. Kidney sizes: right kidney 11.3×5.1x5x2 cm, left kidney 12.6×4.2x5x1cm.

**Fig. 1:**
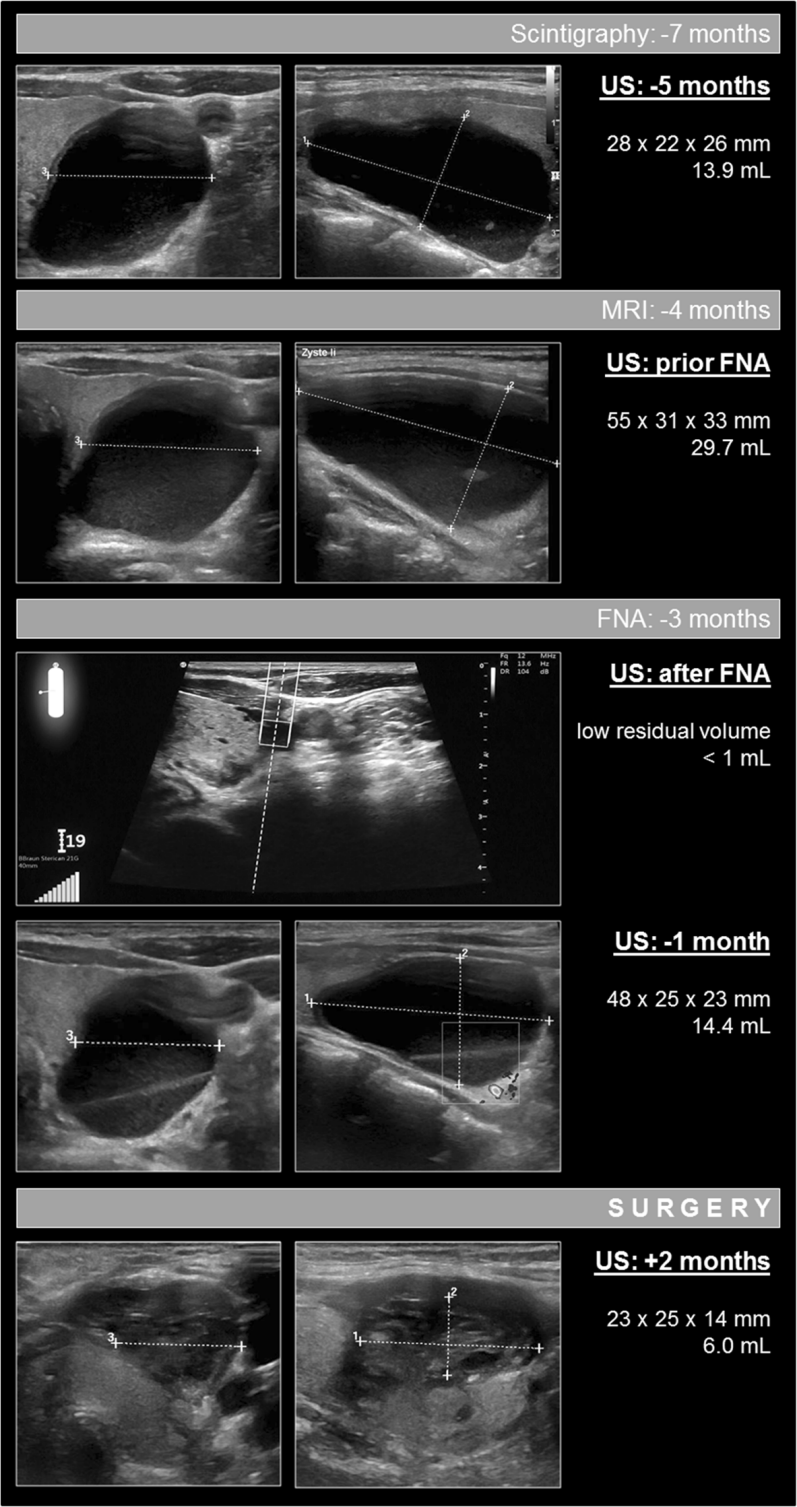
Overview of the diagnostic course. Ultrasonography images of the parathyroid cyst in axial (left image row) and sagittal (right image row) orientations. The last image series shows the post-operative hematoma. Ultrasonography devices used: TOSHIBA Xario, linear probe, 8 MHz; GE LOGIQ S7, linear probe, 8-12 MHz; GE LOGIQ E9, linear probe, 9–15 MHz

**Fig. 2:**
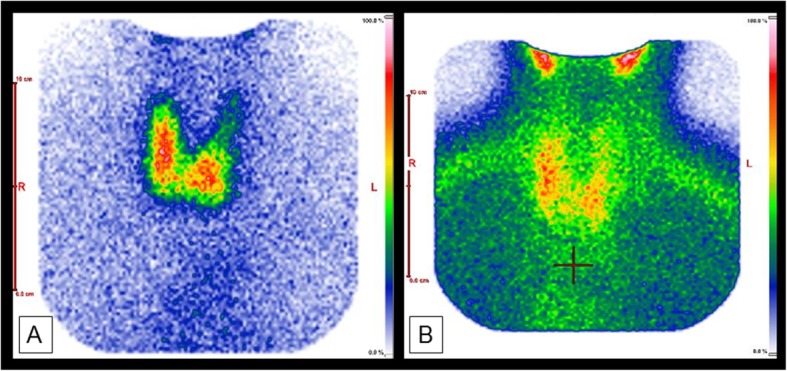
**a** 99m-technetium-pertechnetate scintigraphy: cold spot in correlation to the left-sided cystic lesion. **b** 99m-technetium-MIBI scintigraphy: no pathological tracer uptake of the cystic lesion

Cervical ultrasonography (US) showed a normal thyroid gland (volume: 13 mL) without nodules (Fig. 1). No parathyroid adenoma was found, but a cystic mass (of 10.2 mL), most probably benign, on the left dorsum of the thyroid was identified. 99m-technetium-MIBI scintigraphy failed to find a focus (Fig. 2), especially the assumed cyst did not show pathological tracer uptake. 99m-technetium-pertechnetate scintigraphy revealed reduced left-sided activity in correlation to the cystic lesion. In summary, no obvious parathyroid adenoma could be identified, and the cystic lesion was diagnosed as an unsuspicious thyroidal cyst.

Calcium lowering therapy was performed with forced diuresis and infusion of 5 mg zoledronic acid. Calcium-level decreased to normal values (2.39 mmol/L), but the iPTH remained elevated (100 ng/L). A working diagnosis of primary hyperparathyroidism without detectable focus was made and a therapy with cinacalcet 30 mg/d was initiated. Close ambulatory follow-up in our outpatient department was scheduled.

**Fig. 3:**
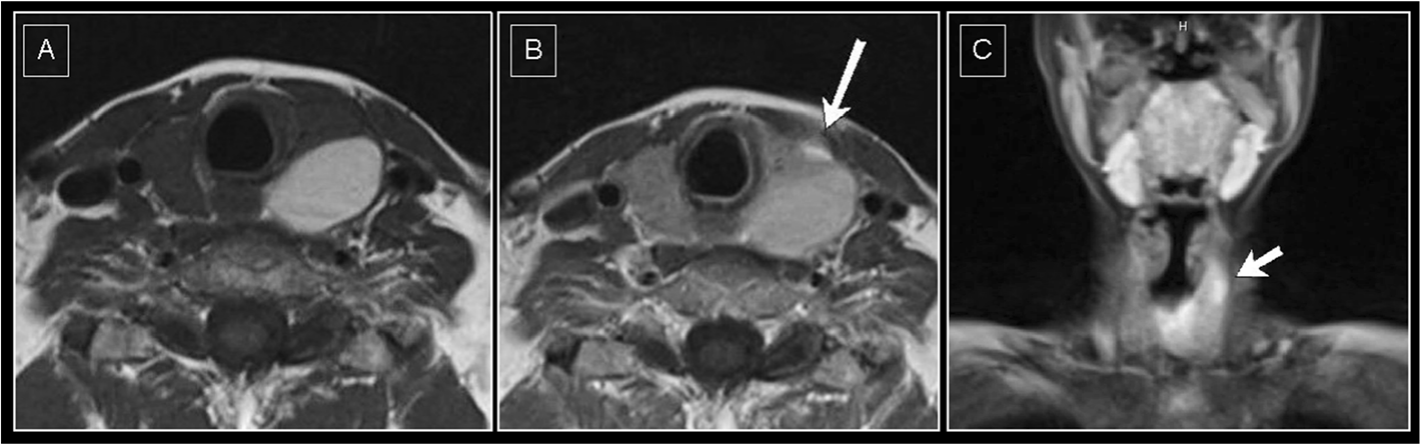
Dynamic contrast agent enhanced MRI: small focal arterial hypervascularization at the ventral part of the capsule of the left-sided parathyroidal cyst. **a** T2w, axial orientation (**b**) T2w, axial orientation, the white arrow indicates focal arterial contrast agent enhancement (**c**) T2w coronal orientation, the white arrow indicates focal arterial contrast agent enhancement

After 2 months of ongoing cinacalcet therapy, serum-calcium increased again to 2.94 mmol/L along with an iPTH of 190 ng/L and a vitamin D (1.25-OH) over 360 pmol/L. The patient had no complaints others than back pain. Sonography of the thyroid gland still did not reveal any further lesions, but the volume of the “cyst” had increased to 13.9 mL. For further clarification, dynamic magnetic resonance angiography of the neck was performed, knowing its high diagnostic accuracy in this field [4]. The examination revealed a small focal arterial hypervascularization at the ventral part of the lesions capsule, which is frequently seen in parathyroidal adenomas (Fig. 3). Nevertheless, since no further suspicious features could be identified on the MRI scan, the images were finally interpreted to be more likely for an unsignificant cystic lesion. Because of the highly elevated calcium level, the dose of cinacalcet was increased to 60 mg/d.

Four months later the patient presented again. She reported a painless swelling on the left site of the neck without pressure symptoms. Despite of the elevated cinacalcet dosage, calcium (2.71 mmol/L) was still elevated, iPTH was 138 ng/L, and the eGFR (CKD-EPI) 84 mL/min. The “cyst” showed further growth to 29.7 mL. Due to patients discomfort and for further diagnostic approach we decided to perform a fine-needle aspiration (FNA), which was carried out with a novel ultrasound-guided standard needle magnetization guidance system [5]. This system enables the needle tip to be tracked, which facilitates the complete aspiration of the fluid and punctation of the wall.

**Fig. 4:**
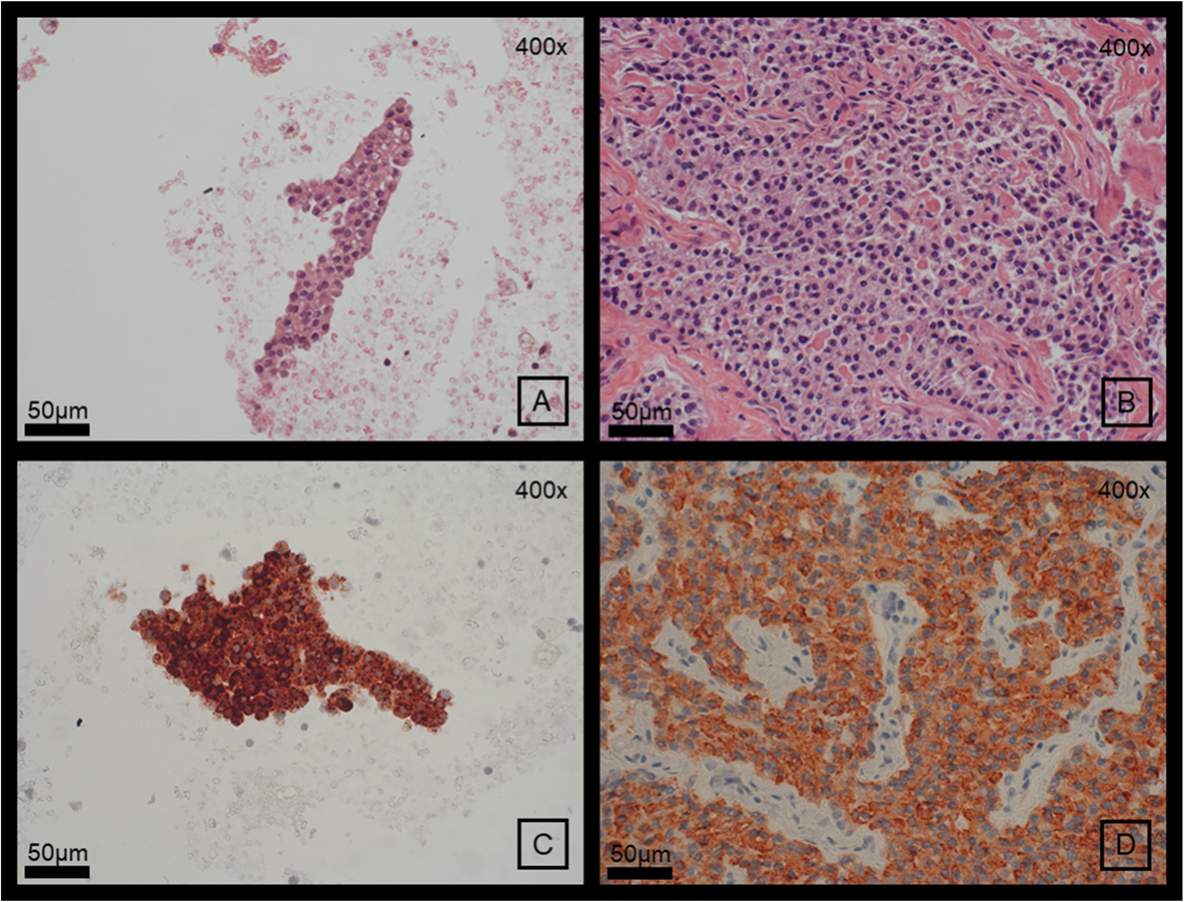
H&E (=hematoxylin and eosin) staining, magnification 400x, of (**a**) cytoblock of material gained by puncture of the cystic adenoma (**b**) the surgical specimen of the adenoma; Immunhistochemical staining for parathormone, counterstaining with hematoxylin, magnification 400x of (**c**) cytoblock of material gained by puncture of the cystic adenoma (**d**) the surgical specimen of the adenoma

The aspirated fluid (approx. 25 mL) was brown at the beginning and slightly bloody at the end. We measured iPTH in the cyst fluid, which was on the upper scale of the calibration curve of the assay (> 1800 ng/L). Dilution (1:100) showed the same result (expected concentration > 180.000 ng/L). Cytopathological workup of the aspirated fluid revealed follicular epithelial cells. Staining for thyroid transcription factor 1 (TTF-1) as well as thyroglobulin (Tg) was negative, suggesting other than thyroid origin. A subsequent immunohistochemical staining for parathormone was positive (Fig. 4, antibody see S1, cytopathological methods see S2).

After the diagnosis was ascertained the cyst was surgically removed 8 months after the initial presentation of the patient. Preoperative sonography revealed that the cyst again had grown from about 5 mL to 14.4 mL about 1 month after FNA. iPTH intraoperatively dropped from 88.7 ng/L to 10.6 ng/L after resection. Intraoperative frozen section as well as routine histopathological workup confirmed diagnosis of cystic parathyroid adenoma. Immunohistochemistry showed strong expression of parathyroidal hormone (Fig. 4).

The patient experienced hypocalcemia on the first postoperative day (1.8 mmol/L), but values quickly normalized under mid-dose oral calcium substitution (300 mg calcium ions per day). In the 2 month follow- up visit serum calcium (2.62 mmol/L) and iPTH (16.6 ng/L) were normalized without further medication. Sonography revealed only a small residual haematoma at the site of the former cyst (Fig. 1).

## Discussion and conclusions

The novelty of our case report is the probable triggering of the cystic growth by cinacalcet. Retrospectively reviewed the parathyroidal cyst was already present in a CT-scan performed due to mamma-carcinoma staging about 4 years before which had a volume of 1.5 mL. As calcium was in the normal range at that timepoint, it was considered as asymptomatic thyroid cyst and iPTH was not measured. The total volume increased slowly by about 8 mL over following 3 years without treatment, but by 21 mL after initiation of cinacalcet. We did not find any case reports or animal trials considering this issue. Only for secondary hyperparathyroidism in hemodialysis patients, adenoma growths as well as regression induced by cinacalcet are described [6]. However, since renal function was normal and serum phosphate low in our patient, we assumed primary hyperparathyroidism. Histopathological changes including haemorrhagic changes, haemosiderin deposition and cystic degeneration are described as well. The latter was seen only in areas with nodular hyperplasia [7]. Known molecular mechanisms mediating volume reduction of adenomas as well as regressive alterations are antiproliferative effects on the parathyroideal cells caused by calcium-sensing-receptor activation as well as induction of apoptosis [6].

Taking this into account, growth induction of the cyst in this case may be explained through cincalcet-induced increased apoptosis of parathyroidal cells.

Yamada et al. [6] also observed a strong association between treatment failure (insufficient reduction of parathormone levels by cinacalcet) and growth instead of volume reduction of the parathyroid adenomas in secondary hyperparathyroidism. Taking this into account, also a treatment failure of cinacalcet in the presented case could explain the further expansion of the cystic adenoma.

We conclude that a cystically degenerated parathyroidal adenoma is a rare entity which should be considered in lack of other foci in primary hyperparathyroidism. Especially in cases with cystical growth and ongoing therapy with cinacalcet, aspiration of cystical fluid and measurement of iPTH in the cystic fluid should be considered early to establish the diagnosis.

## Supplementary information

**Additional file 1: S1.** Laboratory methods.

**Additional file 2: S2**. Method of immunhistochemistry.

## Data Availability

Data on this case not included in this publication are available from the corresponding author on reasonable request.

## Abbreviations

CKD-EPI: Chronic kidney disease epidemiology collaboration equation
eGFR: Estimated glomerular filtration rate
FNA: Fine needle aspiration
iPTH: Intact parathyroidal hormone
MRI: Magnetic resonance imaging
Tg: Thyroglobulin
TSH: Thyroid stimulating hormone
TTF-1: Thyroid transcription factor 1
