# Supplementary material for: Case report of a cystic parathyroidal adenoma with rapid growth induced by cinacalcet
Source: BMC Endocr Disord. 2020 Apr 20;20:53. doi: 10.1186/s12902-020-0532-7 (PMC7171747; doi:10.1186/s12902-020-0532-7)
Supplement: Supplementary file 1 — Additional file 1: S1. Laboratory methods. [file 12902_2020_532_MOESM1_ESM.docx]

**S1** **Laboratory methods**

Plasma-parathormone was measured using LIAISON® N-TACT® PTH II Assay (DiaSorin, Saluggia, Italy).

Antibody against parathormone: rabbit monoclonal [EPR8481] to parathormone (ab166632; Abcam, Cambridge, UK).
