# Supplementary material for: Case report of a cystic parathyroidal adenoma with rapid growth induced by cinacalcet
Source: BMC Endocr Disord. 2020 Apr 20;20:53. doi: 10.1186/s12902-020-0532-7 (PMC7171747; doi:10.1186/s12902-020-0532-7)
Supplement: Supplementary file 2 — Additional file 2: S2. Method of immunhistochemistry. [file 12902_2020_532_MOESM2_ESM.docx]

**S2 Method of immunhistochemistry**

Cytoblock: Material from puncture of the cyst was centrifuged at 3,500 x g for 10 min. Subsequently, 1 mL of pooled human plasma was added to the sample. After brief vortexing, 100 µL fibrinogen was added and the sample was vortexed again. The resulting clots were incubated for 24 h in 10% buffered formalin and then embedded in paraffin blocks.

From the cytoblock and from the paraffin embedded adenoma tissue specimen 4 µm sections were prepared and floated onto positively charged slides. Immunostaining was performed by an indirect peroxidase labeling method as described previously (5). Briefly, sections were dewaxed, microwaved in 10 mM citric acid (pH 6.0) for 16 min at 600 W and then incubated with the specific primary antibody overnight at 4°C. Detection of the primary antibody was performed using a biotinylated anti-rabbit IgG, respectively, followed by an incubation with peroxidase-conjugated avidin (Vector ABC “Elite” kit; Vector, Burlingame, CA). Binding of the primary antibody was visualized using 3-amino-9-ethylcarbazole (AEC) in acetate buffer (BioGenex, San Ramon, CA). Sections were then rinsed, counterstained with Mayer’s hematoxylin (Sigma-Aldrich Chemie GmbH, Steinheim, Germany) and mounted in Vectamount™ mounting medium (Vector Laboratories, Burlingame, CA).
